# Primary lateral sclerosis plus parkinsonism: a case report

**DOI:** 10.1186/s12883-023-03360-x

**Published:** 2023-08-29

**Authors:** Abhaya Moturu, Wade Welch

**Affiliations:** 1 Department of Neurology, University of Kansas St. Francis Health System, Topeka, KS USA; 2 Kansas City University, Kansas City, MO USA

**Keywords:** Primary lateral sclerosis, Parkinsonism, Upper motor neuron symptoms, Pyramidal features, Extrapyramidal features

## Abstract

**Background:**

The standard of diagnosing primary lateral sclerosis, the Pringle criteria, requires three years of purely upper motor neuron symptom presentation before confirming diagnosis. This classic standard has been questioned on occasion due to its restrictive range of both time period and symptomatic exhibition.

**Case presentation:**

This case report will review a 57-year-old Caucasian female who presented with pyramidal and extrapyramidal features suggestive of the exceedingly rare disease primary lateral sclerosis plus parkinsonism. We will describe the mixture of upper motor neuron signs and striking parkinsonian symptoms experienced by the patient, as well as the full diagnostic workup leading to her preliminary diagnosis. The details of this case will then be utilized to explore the diagnostic criteria of primary lateral sclerosis, as well as to work through the differential of conditions resembling Parkinson’s disease.

**Conclusions:**

The current criteria to diagnose primary lateral sclerosis may be excluding patients with the disease and is an ongoing area of investigation. A thorough differential including other neurodegenerative conditions is necessary to consider and requires long-term follow-up.

**Supplementary Information:**

The online version contains supplementary material available at 10.1186/s12883-023-03360-x.

## Background

Primary lateral sclerosis (PLS), accounting for only 3%-5% of motor neuron disease [1] is distinguished by upper motor neuron (UMN) involvement which remains largely exclusive of the lower motor neuron over time. PLS has been reported to have variants depending on the distribution of initial involvement as ascending paraparesis, hemiparetic, or bulbar onset [2]. Increasingly, cases with extra motor neuron manifestations labeled PLS-plus are recognized to include parkinsonism. The heterogeneity of this disease presentation coupled with the absence of robust biomarkers makes diagnosis exceptionally challenging. The rarity of the disease adds an additional layer of complexity, as there is limited data and research available to guide a proper diagnosis.

The presence of UMN symptoms does authorize concern for amyotrophic lateral sclerosis, as this disease can present with UMN involvement prior to lower motor neuron (LMN) involvement [3]. This has led to a deindividualization of PLS that prevents its pathophysiology from being examined as an independent disease process. The current standards of diagnosis require years before confirmation, which inevitably limits a patient's prognosis by delaying essential medical intervention and potential treatment [3]. An improved perspective into the different ways PLS may present is essential to better comprehend this disease for optimal diagnostics and management.

## Case presentation

We describe a 57-year-old right-handed female presenting to a neurology outpatient clinic with a chief complaint of weakness. She initially began to experience hypophonia, weakness and subsequent difficulty walking approximately one year prior, with hoarseness of speech starting even earlier. Initial onset of symptoms also involved bradykinesia, including speech and ambulation. She noted that her left upper and lower extremities were predominantly affected at onset, with subsequent spread to the right upper and lower extremities. In particular, she had difficulty typing, especially with her left hand, and over time developed micrographia with somewhat illegible handwriting. She experienced impaired gait and balance with a tendency to fall backwards and reported difficulty lifting her legs getting in and out of vehicles. She also endorsed stiffness and cramping in her legs.

Review of systems was notable for vague lightheadedness, which she described as a "fuzzy feeling" or slowed mental processing. She had a history of constipation with infrequent bowel movements for several years and exhibited frequent episodes of yelling out at night for which the patient was amnestic. Remarkably, within about six months of disease onset she developed laughing involuntarily out of context. Throughout her symptomatic progression, she did not experienced fevers, chills, or other constitutional symptoms. Past medical history was limited to a diagnosis of shingles several years earlier without residual symptoms, and family history was negative for neurologic disorders including motor neuron disease and movement disorders.

Physical exam included normal orthostatics. Hypomimia was observed with no abnormalities of eye movements. Cognition was normal by Montreal Cognitive Assessment, but speech was hypophonic, slow and mildly dysarthric with hoarse monotone quality. Jaw jerk was absent, and there was no evidence for tongue atrophy or fasciculations. Power was normal without muscle atrophy or fasciculations. Hyperreflexia with Hoffmann's and suprapatellar reflexes were noted bilaterally. Mild bilateral lower extremity spasticity was slightly greater on the left with bilateral extensor plantar responses present. Gait was slow en bloc with bilaterally decreased arm swing. Mild bradykinesia was seen in the upper extremities but without cogwheeling or resting tremor appreciated. Subsequent further evaluation showed severely impaired finger tapping bilaterally and moderate postural instability.

**Fig. 1:**
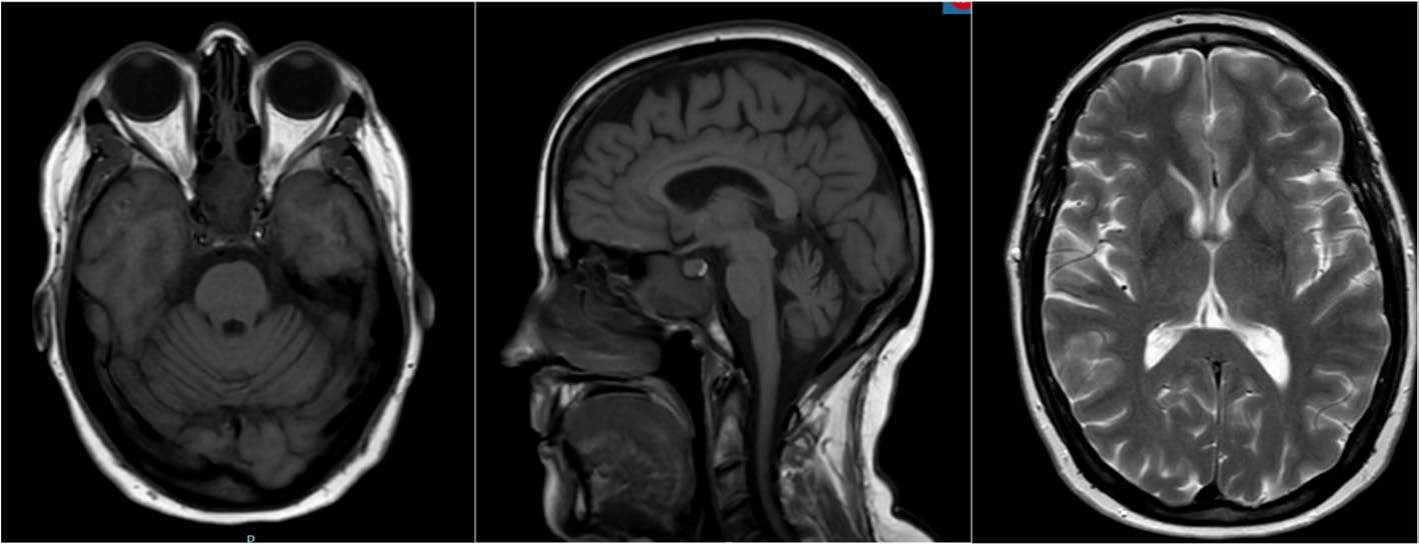
MRI brain imaging nine months after symptomatic onset. Left to right–T1 axial view; T1 sagittal view; T2 axial view

**Fig. 2:**
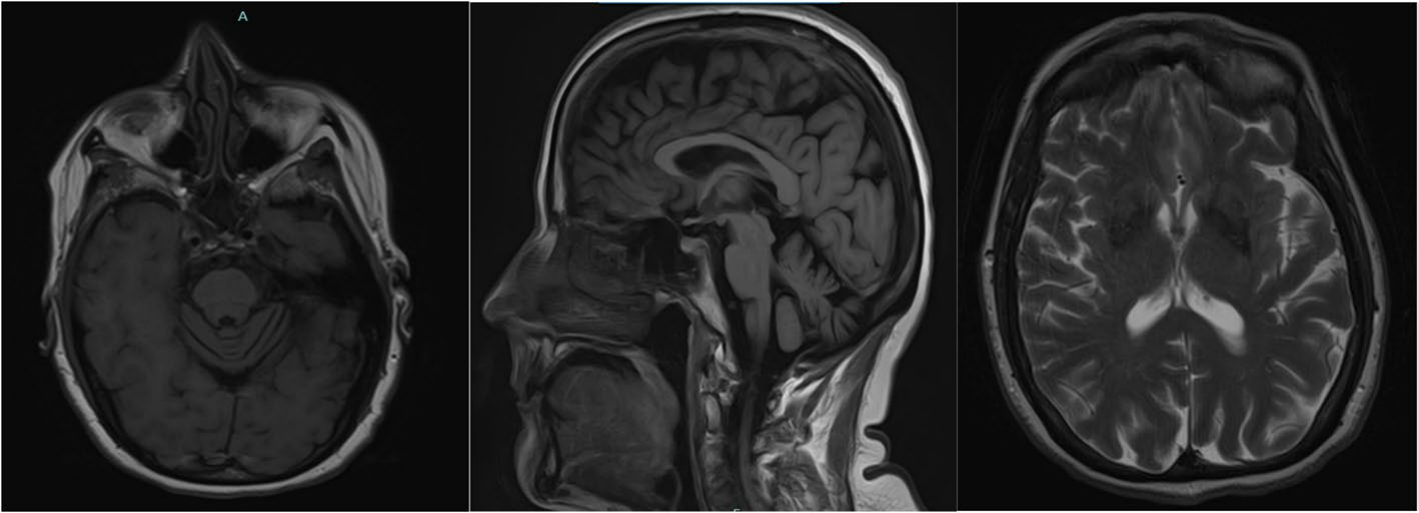
MRI brain imaging two years after symptomatic onset. Left to right–T1 axial view; T1 sagittal view; T2 axial view

**Fig. 3:**
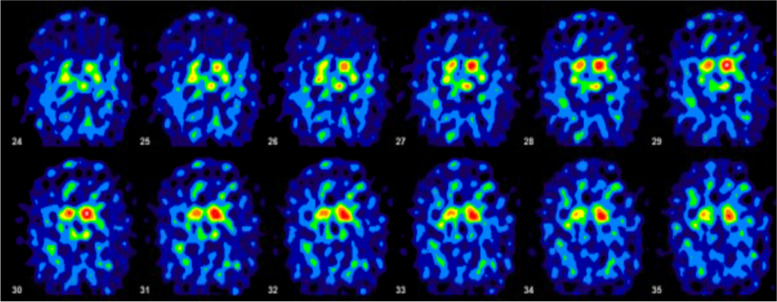
DaTscan (1–123 ioflupane) one year after symptomatic onset

Laboratory studies including routine blood work and other testing were negative for autoimmune and paraneoplastic processes, vitamin deficiency, and metabolic disorders (Table 1). Genetic analysis revealed a variant of uncertain significance in the PSEN2 gene (Table 2). Electromyography was completely negative for lower motor neuron findings. Magnetic resonance imaging (MRI) of the brain both at nine months and two years from disease onset revealed atrophy mildly greater than expected for the patient’s age, particularly in the frontal and parietal cortices bilaterally symmetrical with a normal-appearing brainstem, including pons. Neither a hot cross bun nor putaminal rim sign was visualized (Figs. 1 and 2). MRI of the cervical spine was essentially normal with minimal disc bulging and no intrinsic cord findings. A DaTscan roughly one year from symptom onset was markedly abnormal with no activity in the putamen bilaterally and diminished uptake in the caudate bilaterally, moderate on the right and mild on the left, confirming nigrostriatal dysfunction (Fig. 3).

**Table 1:** Laboratory values

Component	Result	Reference Range
Sedimentary Rate	10 mm/hr	1—20 mm/hr
C-Reactive Protein	< 2.9 mg/L	< 3.1 mg/L
Total Creatine Kinase	73 U/L	26—192 U/L
IgA	219	70—400 mg/dL
Total IgG	1080	700—1600 mg/dL
IgM	183	40—230 mg/dL
Serum Immunofixation	No paraprotein seen	N/A
Total Protein	7.6	N/A
Albumin, Electrophoresis	3.8	3.5—5.0 g/dL
Albumin %	49.8	48.1—59.5%
Alpha 1	0.3	0.1—0.3 g/dL
Alpha 1%	3.7	2.3—4.9%
Alpha 2	0.9	0.5—1.2 g/dL
Alpha 2%	11.2	6.9—13.0%
Beta Globulin	1.3 g/dL	0.8—1.3 g/dL
Beta %	17.4%	13.8—19.7%
Gamma	1.4 g/dL	0.6—1.7 g/dL
Gamma globulin %	17.9%	10.1—21.9%
A/G Ratio	1.0	1.0—1.8
S SA 52 (RO) (ENA) Antibody, IgG	10 AU/mL	0—40 AU/mL
S SA 60 (RO) (ENA) Antibody, IgG	1 AU/mL	0—40 AU/mL
Sjogren SSB Antibody (LA)	10 AU/mL	0—40 AU/mL
ANA, IgG by ELISA	None detected	N/A
ENA Smith Antibody	4 AU/mL	0—40 AU/mL
Purkinje Cell/Neuronal Nuclear IgG Screen	None detected	N/A
MMA Serum/Plasma, Metabolic Disorder	0.10 umol/L	0.00—0.40 umol/L
TSH	2.83 mcu/mL	0.35—5.00 mcu/mL
Copper	1.12 mcg/mL	0.75—1.45 mcg/mL
Vitamin B12	515	180—914 pg/mL
Methylmalonic Acid	0.09 umol/mL	0.00 – 0.40 umol/mL

**Table 2:** Genetic testing

Disease Panel	Abnormal Gene	Variant	Zygosity	Variant Classification	Interpretation
Amyotrophic Lateral Sclerosis	C9orf72	Hexanucleotide Repeat Units Detected	Homozygous	Normal range	Normal range repeats not associated with disease
Hereditary Amyotrophic Lateral Sclerosis, Frontotemporal Dementia, Alzheimer Disease	PSEN2	c.442G > A(p.Val148lle)	Heterozygous	Uncertain significance	PSEN2 associated with autosomal dominant AD4, and variant missense not associated with change in gene function; not enough evidence currently to determine significance
Comprehensive Neuropathies, Comprehensive Hereditary Spastic Paraplegia Comprehensive	No abnormal genes in 170-gene panel	N/A	N/A	N/A	No pathogenic variants associated with disease
Hereditary Parkinson Disease and Parkinsonism	No abnormal genes in 29-gene panel	N/A	N/A	N/A	No pathogenic variants associated with disease

Follow-up 2.5 years after initial symptom onset, the patient’s clinical course has been a very slow progression. She continues to have pathologically brisk reflexes with spasticity limiting functionality and increased tone impacting her dexterity and gait. Neuropsychological testing reveals normal cognition with exception of mild slowing in verbal construction and cognitive processing. Ocular motor function remains normal. She continues to exhibit mild asymmetry with both spasticity and bradykinesia slightly worse on the left, still without tremor. She ambulates independently and functions well in her job though challenged by hypophonia and difficulty typing. She has not developed orthostatic hypotension or other signs of autonomic failure. Treatment with tizanidine has helped symptoms of stiffness and cramping, and her bradykinesia has subjectively responded to levodopa titrated up to tolerance.

## Discussion and conclusions

Our patient has exhibited clear clinical features for upper motor neuron dysfunction. The speech and upper extremity involvement effectively excludes hereditary spastic paraplegia (HSP). Central nervous system structural or demyelinating disease was not evident on MRI studies. Infectious etiology appears unlikely given the absence of constitutional symptoms, slow course of illness, and geographic factors. Amyotrophic lateral sclerosis must be considered in the differential diagnosis, as cases of late transition of upper motor neuron disease to ALS have been described typically between three to four years [3]. Historically, symptom duration of three years proposed in the 1992 Pringle criteria was required for PLS diagnosis. Subsequently, the 2006 Gordon criteria proposed a cutoff of four years [1], supported as an outside boundary based on a larger Mayo Clinic study population in which none of the patients developed ALS after this time period [2]. Delay in diagnosis hinders research efforts to better understand the disease process and places a burden on patients. To expedite diagnosis, a 2020 consensus statement established a new category of “probable PLS” requiring a shorter symptom duration of two to four years [3].

Our patient exhibited extrapyramidal features including prominent bradykinesia and postural instability but with atypical features of early dysarthria, absence of resting tremor, and limited response to L-dopa, suggesting a diagnosis of atypical parkinsonism rather than primary Parkinson's disease. Corticobasal degeneration (CBD) would be another consideration but is distinguished by somewhat unique cortical signs, marked asymmetry, and rapid clinical course not consistent with our patient's presentation. Progressive supranuclear palsy (PSP) would be unlikely without eye movement abnormalities, and due to the patient’s ongoing normal ocular motor function, this condition remains lower on our differential currently. The patient will continue to be monitored for any corresponding changes that may indicate PSP over PLS-P.

Multiple system atrophy (MSA) has subtypes including MSA plus parkinsonism (MSA–P) [4, 5], which can be difficult to distinguish from primary lateral sclerosis plus parkinsonism (PLS-P). Corticospinal signs are present in 40%-50% of patients with MSA [6], but autonomic failure is a key component of this diagnosis. Our patient did report chronic mild constipation and frequent screaming out at night possibly reflective of REM sleep behavior disorder (RBD), and both are common antecedents for Parkinson syndromes. She also reported urinary frequency over several years, but bladder instability is thought to also be common in PLS [3]. Although she has mentioned a sense of dizziness, orthostatics have been consistently normal. MRI findings lacked the “hot cross bun” sign and the “putaminal slit” indicative of MSA, with the latter finding specifically associated with MSA-P [7]. These findings, along with the patient’s lack of autonomic involvement discourages MSA as a potential cause for her clinical presentation. Interestingly, she did develop a pseudobulbar affect with inappropriate laughing, which is commonly seen in PLS patients [1], but may also be seen in ALS, parkinsonism, and other neurodegenerative conditions.

The true incidence of overlap syndromes with motor neuron disease and parkinsonism is unknown. One study showed that in a cohort of 1042 motor neuron disease patients, 18 patients were diagnosed with parkinsonian syndromes [8]. This shared phenotype was far more common with PLS than other motor neuron disease subtypes, and atypical parkinsonian syndromes predominated with PLS, fitting our case. In this cohort, DaTscan data when available revealed bilateral asymmetric abnormalities similar to our case in two thirds of both PLS and ALS patients [8]. Others have reported frontal cortex hypometabolism with fluorodeoxyglucose positron emission tomography (FDG-PET) scans in patients with parkinsonism in the absence of nigrostriatal involvement [9]. One case report also demonstrated hypometabolism in the sensorimotor cortex contralateral to pyramidal signs in a patient with asymmetric upper motor neuron dysfunction and mild parkinsonism [10]. This has led to speculation that widespread frontal lobe impairment could be responsible for extrapyramidal features [9], which is plausible given the rich network connectivity between the frontal lobe and basal ganglia.

Nevertheless, the pathogenesis for PLS and PLS-plus syndromes remains largely elusive. Genetic testing was not diagnostic in our case but has been useful in parsing out HSP from PLS with spastic paraparetic onset, and testing for C9orf72 expansion may identify familial ALS (Table 2). Neuronal and glial TDP-43 cytoplasmic inclusions are seen in a majority of ALS patients [3] and have been seen with PLS [1]. There was a report of ubiquitinated neuronal inclusions seen in the frontal and temporal cortex of a patient presenting as primary lateral sclerosis and parkinsonism [11]. However, a unifying histopathological picture has not emerged from postmortem studies [3].

It is of interest that genetic screening revealed a rare variant in the PSEN2 gene. PSEN2 mutations have been known to be pathogenic in familial Alzheimer's disease but have increasingly been implicated in other disorders including Parkinson's disease with dementia [12]. This patient’s missense variant remains a variant of uncertain significance according to the latest ACMG criteria. However, recent data indicates that PSEN2 may have a stronger correlation to general neurodegeneration [12]. Additionally, one case report describes the presence of PSEN1 in a patient with both early onset familial Alzheimer’s disease and confirmed PLS [13], providing a hint that PSEN genes may contribute to PLS pathogenesis.

Newer imaging strategies may pave the way for enhanced understanding and diagnosis of PLS.

A prospective study incorporated the 2020 consensus criteria to define probable PLS and compared this group with established PLS patients using structural and diffusion MRI [14], with findings suggestive for early involvement of gray matter followed by development of white matter changes. They state that this is the opposite of the sequence seen in ALS in which early subcortical corticospinal tract and corpus callosum involvement precedes later cortical degeneration. This is an intriguing finding which could shed light on how the timing and extent of lower motor neuron involvement differs along the spectrum between PLS and ALS. Additionally, none of the probable PLS patients in the study went on to develop ALS, helping to validate the shorter timeframe for diagnosis proposed in the consensus diagnostic criteria [12]. In motor neuron disease, FDG-PET scans may show the stripe sign, indicating hypometabolism in the primary motor cortex, but imaging cannot distinguish PLS from ALS [15] and is not included in the 2020 PLS consensus diagnostic criteria [3].

FDG-PET also appears promising in differential diagnosis for Parkinson’s disease and atypical Parkinson’s syndromes, but further studies using postmortum verification of diagnosis may be required for validation [16, 17]. PET scan tracers with individual molecular targets are being developed to improve diagnostic specificity, such as an antibody-based PET scan for imaging degenerative pathology related to alpha-synuclein deposition [18]. Recent data presented at the AD/PD conference in March 2022 demonstrated selective binding of a novel tracer to the cerebellar white matter and peduncles of patients with MSA, but not in other synucleinopathies such as Parkinson's disease and dementia with Lewy bodies [19]. This could prove to be helpful in our case by distinguishing MSA from PLS–P.

There is a critical need for better understanding of the pathogenesis of this disease and its classification, complicated by subtypes sharing many symptoms distinguished more by magnitude than uniqueness. Delayed diagnosis in addition to impeding research prolongs patient fear of developing ALS, could prevent access to proper rehabilitation [20] and may lead to improper diagnosis [21]. More certain diagnosis and prognosis empowers patients with knowledge that enables increased participation in their treatment plan as well as research trials.

Greater multicenter and international collaboration will likely be necessary to pool large enough patient numbers necessary to identify diagnostic and predictive markers meaningful in PLS.

Overall, this case report provides an understanding of how PLS-P may present and how it can be differentiated from other neurodegenerative conditions with Parkinson-like symptoms.

It exhibits the richness of an overlap syndrome which appears to be part of the continuum of both motor neuron disease and parkinsonism. It is still too early to exclude the possibility for later transition to ALS, but our patient’s clinical course with slow progression of exclusively upper motor neuron dysfunction is more consistent with the PLS phenotype. The absence of MSA signature abnormalities on MRI two years into the disease along with below threshold autonomic dysfunction argues against MSA-P, the chief alternant diagnosis under consideration. We look forward to validation of imaging or other biochemical biomarkers which would provide a more conclusive diagnosis.

### Supplementary Information

**Additional file 1:** **Supplementary Table 1.** Invitae Amyotrophic Lateral Sclerosis with C9orf72 Panel.

**Additional file 2:** **Supplementary Table 2.** Invitae Hereditary Amyotrophic Lateral Sclerosis, Frontotemporal Dementia and Alzheimer Disease Panel with Add-On Preliminary Evidence Genes.

**Additional file 3:** **Supplementary Table 3.** Invitae Hereditary Parkinson Disease and Parkinsonism Panel with Add-on Preliminary Evidence Genes.

**Additional file 4:** **Supplementary Table 4.** Invitae Comprehensive Neuropathies Panel, Hereditary Spastic Paraplegia Comprehensive Panel, and Add-on Preliminary Evidence Genes.

## Data Availability

Data sharing is not applicable to this article as no datasets were generated or analyzed during the current study.

## Abbreviations

MRI: Magnetic resonance imaging
DaTscan: Dopamine transporter scan
PLS: Primary lateral sclerosis
UMN: Upper motor neuron
HSP: Hereditary spastic paraplegia
ALS: Amyotrophic lateral sclerosis
PSP: Progressive supranuclear palsy
CBD: Corticobasal degeneration
MSA: Multiple system atrophy
MSA-P: Multiple system atrophy plus parkinsonism
PLS-P: Primary lateral sclerosis plus parkinsonism
RBD: REM sleep behavior disorder
FDG-PET: Fluorodeoxyglucose positron emission tomography
AD4: Alzheimer disease type 4
