# Supplementary material for: Primary lateral sclerosis plus parkinsonism: a case report
Source: BMC Neurol. 2023 Aug 29;23:312. doi: 10.1186/s12883-023-03360-x (PMC10463512; doi:10.1186/s12883-023-03360-x)
Supplement: Supplementary file 1 — Additional file 1: Supplementary Table 1. Invitae Amyotrophic Lateral Sclerosis with C9orf72 Panel. [file 12883_2023_3360_MOESM1_ESM.docx]

Supplementary Table 1: Invitae Amyotrophic Lateral Sclerosis with C9orf72 Panel

|  | |
| --- | --- |
| **Gene** | **Transcript Reference** |
| ALS2 | NM_020919.3 |
| ANG | NM_001145.4 |
| ANXA11 | NM_001157.2 |
| C9orf72 | NM_001256054.2 |
| CHCHD10 | NM_213720.2 |
| DCTN1 | NM_004082.4 |
| ERBB4 | NM_005235.2 |
| FUS | NM_004960.3 |
| HEXA | NM_000520.4 |
| KIF5A | NM_004984.2 |
| OPTN | NM_021980.4 |
| PFN1 | NM_005022.3 |
| SETX | NM_015046.5 |
| SOD1 | NM_000454.4 |
| SPG11 | NM_025137.3 |
| SQSTM1 | NM_003900.4 |
| TARDBP | NM_007375.3 |
| TBK1 | NM_013254.3 |
| TFG | NM_006070.5 |
| UBQLN2 | NM_013444.3 |
| VAPB | NM_004738.4 |
| VCP | NM_007126.3 |
