# Supplementary material for: Primary lateral sclerosis plus parkinsonism: a case report
Source: BMC Neurol. 2023 Aug 29;23:312. doi: 10.1186/s12883-023-03360-x (PMC10463512; doi:10.1186/s12883-023-03360-x)
Supplement: Supplementary file 2 — Additional file 2: Supplementary Table 2. Invitae Hereditary Amyotrophic Lateral Sclerosis, Frontotemporal Dementia and Alzheimer Disease Panel with Add-On Preliminary Evidence Genes. [file 12883_2023_3360_MOESM2_ESM.docx]

Supplementary Table 2. Invitae Hereditary Amyotrophic Lateral Sclerosis, Frontotemporal Dementia and Alzheimer Disease Panel with Add-On Preliminary Evidence Genes

| **Gene** | **Transcript Reference** |
| --- | --- |
| ALS2 | NM_020919.3 |
| ANG | NM_001145.4 |
| ANXA11 | NM_001157.2 |
| APP | NM_000484.3 |
| ATP13A2 | NM_022089.3 |
| CHCHD10 | NM_213720.2 |
| CHMP2B | NM_014043.3 |
| DCTN1 | NM_004082.4 |
| DDHD1 | NM_001160147.1 |
| ERBB4 | NM_005235.2 |
| ERLIN1 | NM_006459.3 |
| FIG4 | NM_014845.5 |
| FUS | NM_004960.3 |
| GRN | NM_002087.3 |
| HEXA | NM_000520.4 |
| HNRNPA2B1 | NM_031243.2 |
| ITM2B | NM_021999.4 |
| KIF5A | NM_004984.2 |
| LRRK2 | NM_198578.3 |
| MAPT | NM_005910.5 |
| MATR3 | NM_199189.2 |
| NEFH | NM_021076.3 |
| OPTN | NM_021980.4 |
| PFN1 | NM_005022.3 |
| PRNP | NM_000311.3 |
| PSEN1 | NM_000021.3 |
| PSEN2 | NM_000447.2 |
| SETX | NM_015046.5 |
| SIGMAR1 | NM_005866.3 |
| SNCA | NM_000345.3 |
| SOD1 | NM_000454.4 |
| SORL1 | NM_003105.5 |
| SPG11 | NM_025137.3 |
| SQSTM1 | NM_003900.4 |
| TARDBP | NM_007375.3 |
| TBK1 | NM_013254.3 |
| TFG | NM_006070.5 |
| TIA1 | NM_022173.2 |
| TREM2 | NM_018965.3; NM_001271821.1 |
| UBQLN2 | NM_013444.3 |
| VAPB | NM_004738.4 |
| VCP | NM_007126.3 |
