# Supplementary material for: Primary lateral sclerosis plus parkinsonism: a case report
Source: BMC Neurol. 2023 Aug 29;23:312. doi: 10.1186/s12883-023-03360-x (PMC10463512; doi:10.1186/s12883-023-03360-x)
Supplement: Supplementary file 3 — Additional file 3: Supplementary Table 3. Invitae Hereditary Parkinson Disease and Parkinsonism Panel with Add-on Preliminary Evidence Genes. [file 12883_2023_3360_MOESM3_ESM.docx]

Supplementary Table 3: Invitae Hereditary Parkinson Disease and Parkinsonism Panel with Add-on Preliminary Evidence Genes

| **Gene** | **Transcript Reference** |
| --- | --- |
| ATP13A2 | NM_022089.3 |
| ATP7B | NM_000053.3 |
| CHCHD2 | NM_016139.2 |
| CSF1R | NM_005211.3 |
| DCTN1 | NM_004082.4 |
| DNAJC6 | NM_001256864.1 |
| FBXO7 | NM_012179.3 |
| GBA | NM_001005741.2 |
| GCH1 | NM_000161.2 |
| LRRK2 | NM_198578.3 |
| MAPT | NM_005910.5 |
| PARK7 | NM_007262.4 |
| PDE8B | NM_003719.3 |
| PINK1 | NM_032409.2 |
| PLA2G6 | NM_003560.2 |
| PODXL | NM_005397.3 |
| PRKN | NM_004562.2 |
| PRKRA | NM_003690.4 |
| RAB39B | NM_171998.3 |
| SLC6A3 | NM_001044.4 |
| SNCA | NM_000345.3 |
| SPR | NM_003124.4 |
| SYNJ1 | NM_003895.3 |
| TH | NM_199292.2 |
| TMEM230 | NM_001009923.1 |
| UCHL1 | NM_004181.4 |
| VPS13C | NM_020821.2 |
| VPS35 | NM_018206.4 |
| XPR1 | NM_004736.3 |
