# Supplementary material for: Primary lateral sclerosis plus parkinsonism: a case report
Source: BMC Neurol. 2023 Aug 29;23:312. doi: 10.1186/s12883-023-03360-x (PMC10463512; doi:10.1186/s12883-023-03360-x)
Supplement: Supplementary file 4 — Additional file 4: Supplementary Table 4. Invitae Comprehensive Neuropathies Panel, Hereditary Spastic Paraplegia Comprehensive Panel, and Add-on Preliminary Evidence Genes. [file 12883_2023_3360_MOESM4_ESM.docx]

Supplementary Table 4: Invitae Comprehensive Neuropathies Panel, Hereditary Spastic Paraplegia Comprehensive Panel, and Add-on Preliminary Evidence Genes

| **Gene** | **Transcript Reference** |
| --- | --- |
| AARS | NM_001605.2 |
| ABCD1 | NM_000033.3 |
| ADGRB2 | NM_001294336.1 |
| AIFM1 | NM_004208.3 |
| ALDH18A1 | NM_002860.3 |
| ALS2 | NM_020919.3 |
| AMPD2 | NM_001257360.1 |
| AP4B1 | NM_006594.3 |
| AP4E1 | NM_007347.4 |
| AP4M1 | NM_004722.3 |
| AP4S1 | NM_007077.4 |
| AP5Z1 | NM_014855.2 |
| APOA1 | NM_000039.2 |
| ARG1 | NM_000045.3 |
| ARL6IP1 | NM_015161.2 |
| ARSI | NM_001012301.2 |
| ASAH1 | NM_177924.3 |
| ATL1 | NM_015915.4 |
| ATL3 | NM_015459.4 |
| ATP13A2 | NM_022089.3 |
| ATP1A1 | NM_000701.7 |
| ATP2B4 | NM_001001369.2 |
| ATP7A | NM_000052.6 |
| B4GALNT1 | NM_001478.4 |
| BAG3 | NM_004281.3 |
| BICD2 | NM_001003800.1 |
| BSCL2 | NM_032667.6 |
| C12orf65 | NM_152269.4 |
| C19orf12 | NM_001031726.3 |
| CAPN1 | NM_001198868.1 |
| CCT5 | NM_012073.4 |
| CHCHD10 | NM_213720.2 |
| COX6A1 | NM_004373.3 |
| CPT1C | NM_001136052.2 |
| CYP27A1 | NM_000784.3 |
| CYP2U1 | NM_183075.2 |
| CYP7B1 | NM_004820.3 |
| DCTN1 | NM_004082.4 |
| DDHD1 | NM_001160147.1 |
| DDHD2 | NM_015214.2 |
| DHTKD1 | NM_018706.6 |
| DNAJB2 | NM_001039950.1 |
| DNM2 | NM_001005360.2 |
| DNMT1 | NM_001130823.1 |
| DRP2 | NM_001939.2 |
| DST | NM_001723; NM_015548.4 |
| DSTYK | NM_015375.2 |
| DYNC1H1 | NM_001376.4 |
| EGR2 | NM_000399.3 |
| ELP1 | NM_003640.3 |
| ENTPD1 | NM_001776.5 |
| ERLIN1 | NM_006459.3 |
| ERLIN2 | NM_007175.6 |
| EXOSC3 | NM_016042.3 |
| EXOSC9 | NM_001034194.1 |
| FA2H | NM_024306.4 |
| FARS2 | NM_006567.3 |
| FBLN5 | NM_006329.3 |
| FBXO38 | NM_030793.4 |
| FGD4 | NM_139241.3 |
| FIG4 | NM_014845.5 |
| GAN | NM_022041.3 |
| GARS | NM_002047.2 |
| GBA2 | NM_020944.2 |
| GDAP1 | NM_018972.2 |
| GJB1 | NM_000166.5 |
| GJC2 | NM_020435.3 |
| GLA | NM_000169.2 |
| GNB4 | NM_021629.3 |
| GSN | NM_000177.4 |
| HACE1 | NM_020771.3 |
| HARS | NM_002109.5 |
| HEXA | NM_000520.4 |
| HINT1 | NM_005340.6 |
| HMBS | NM_000190.3 |
| HSPB1 | NM_001540.3 |
| HSPB8 | NM_014365.2 |
| HSPD1 | NM_002156.4 |
| IBA57 | NM_001010867.3 |
| IGHMBP2 | NM_002180.2 |
| INF2 | NM_022489.3 |
| KCNA2 | NM_004974.3 |
| KDM5C | NM_004187.3 |
| KIDINS220 | NM_020738.2 |
| KIF1A | NM_004321.6 |
| KIF1C | NM_006612.5 |
| KIF5A | NM_004984.2 |
| KLC2 | NM_022822.2 |
| L1CAM | NM_000425.4 |
| LITAF | NM_004862.3 |
| LMNA | NM_170707.3 |
| LRSAM1 | NM_138361.5 |
| MAG | NM_002361.3 |
| MARS | NM_004990.3 |
| MCM3AP | NM_003906.4 |
| MED25 | NM_030973.3 |
| MFN2 | NM_014874.3 |
| MME | NM_007289.2 |
| MORC2 | NM_001303256.2 |
| MPZ | NM_000530.6 |
| MTMR2 | NM_016156.5 |
| NDRG1 | NM_006096.3 |
| NEFH | NM_021076.3 |
| NEFL | NM_006158.4 |
| NGF | NM_002506.2 |
| NIPA1 | NM_144599.4 |
| NKX6-2 | NM_177400.2 |
| NT5C2 | NM_012229.4 |
| NTRK1 | NM_001012331.1 |
| PDK3 | NM_001142386.2 |
| PGAP1 | NM_024989.3 |
| PLEKHG5 | NM_020631.4 |
| PLP1 | NM_000533.4 |
| PMP2 | NM_002677.3 |
| PMP22 | NM_000304.3 |
| PNPLA6 | NM_006702.4 |
| POLG | NM_002693.2 |
| POLG2 | NM_007215.3 |
| PRDM12 | NM_021619.2 |
| PRPS1 | NM_002764.3 |
| PRX | NM_181882.2 |
| RAB3GAP2 | NM_012414.3 |
| RAB7A | NM_004637.5 |
| REEP1 | NM_022912.2 |
| REEP2 | NM_001271803.1 |
| RETREG1 | NM_001034850.2 |
| RTN2 | NM_005619.4 |
| SACS | NM_014363.5 |
| SBF1 | NM_002972.3 |
| SBF2 | NM_030962.3 |
| SCN11A | NM_014139.2 |
| SCN9A | NM_002977.3 |
| SEPT9 | NM_006640.4 |
| SH3TC2 | NM_024577.3 |
| SIGMAR1 | NM_005866.3 |
| SLC12A6 | NM_133647.1 |
| SLC16A2 | NM_006517.4 |
| SLC25A46 | NM_138773.2 |
| SLC33A1 | NM_004733.3 |
| SLC52A2 | NM_024531.4 |
| SLC52A3 | NM_033409.3 |
| SLC5A7 | NM_021815.2 |
| SMN1 | NM_000344.3 |
| SMN2 | NM_017411.3 |
| SPART | NM_015087.4 |
| SPAST | NM_014946.3 |
| SPG11 | NM_025137.3 |
| SPG21 | NM_016630.6 |
| SPG7 | NM_003119.3 |
| SPTLC1 | NM_006415.3 |
| SPTLC2 | NM_004863.3 |
| SURF1 | NM_003172.3 |
| TECPR2 | NM_014844.3 |
| TFG | NM_006070.5 |
| TRIM2 | NM_001130067.1 |
| TRPV4 | NM_021625.4 |
| TTR | NM_000371.3 |
| UBA1 | NM_003334.3 |
| UCHL1 | NM_004181.4 |
| USP8 | NM_005154.4 |
| VAMP1 | NM_014231.3 |
| VAPB | NM_004738.4 |
| VPS37A | NM_152415.2 |
| VRK1 | NM_003384.2 |
| WASHC5 | NM_014846.3 |
| WNK1 | NM_213655.4 |
| YARS | NM_003680.3 |
| ZFR | NM_016107.3 |
| ZFYVE26 | NM_015346.3 |
| ZFYVE27 | NM_001002261.3 |
